# Time for Inclusion of Racial and Gender Discrimination in Routine Clinical Assessment

**DOI:** 10.1007/s40615-021-01061-0

**Published:** 2021-05-24

**Authors:** Cristina De Rose, Olivia Spinola, Danilo Buonsenso

**Affiliations:** 1 Department of Woman and Child Health and Public Health, Fondazione Policlinico Universitario A. Gemelli IRCCS, Rome, Italy; 2 Department of Dynimic and Clinical Psycology, Sapienza University of Rome, Rome, Italy; 3 Dipartimento di Scienze biotecnologiche di base, cliniche intensivologiche e perioperatorie, Università Cattolica del Sacro Cuore, Roma, Italia; 4 Global Health Research Institute, Università Cattolica del Sacro Cuore, Roma, Italia

**Keywords:** Racism, Gender discrimination, Child abuse, Children, Education, Mental health

## Abstract

Recently, there has been an increasing amount of scientific interest towards the broad theme of racial inequalities and their impact on human health, specifically exploring how ethnic discrimination affects the wellness of black people and the COVID-19 pandemic. Some of these conditions of inequity also affect black children. Discrimination and racism should be routinely considered as causative agents or triggers of disease and routinely included in clinical examination, during history collection and evaluation of vital signs. This will benefit child and family health, worldwide. We shared our recent experience by reporting a case of a 13-year-old black girl who came to Italy from Niger about 3 years earlier through the traumatizing migratory journey. She was evaluated in the Pediatric Emergency Department (PED) for sleepiness that had progressively worsened during the last days. We describe the case and how it was handled differently by pediatricians and pediatric trainees with equally different personal and professional backgrounds.

We also report the preliminary results of a national survey aimed to assess discrimination and inequalities in Italian Paediatric Residency Schools. Medical ability has been allowing us to respond rapidly to a novel virus in order to save lives. The expertise of doctors and researchers must be used to evaluate this hidden crisis as well, to address racism and injustice and to protect vulnerable people from harm. Our case showed us how it is essential including racial and gender discrimination in a diagnostic process.

**Supplementary Information:**

The online version contains supplementary material available at 10.1007/s40615-021-01061-0.

Following the death of George Floyd, hundreds of thousands of people - initially in the U.S. and then all over the world - started pouring into the streets to demonstrate their dissent and opposition against the growing episodes of racism and discrimination. Even Italy has been witnessing a rise in protests against racism. As declared by a 24-year-old black boy, an actor among the few blacks who could access the Experimental Center of Cinematography in Rome during one of the protest rallies in Rome on June 2020, “if you are a black boy in Italy every morning, when you wake up, you know you have to prove something.”

Daily racism against black people, forever overt in the USA, also now plagues Italy, especially due to the intensification of immigration in recent years. This is plainly apparent in schools, workplaces, and on the street.

Recently, there has been an increasing amount of scientific interest towards the broad theme of racial inequalities and their impact on human health, specifically exploring how ethnic discrimination affects the wellness of black people and the COVID-19 pandemic [1–3]. Some of these conditions of inequity also affect black children. They are characterized, just to cite a few, by inequalities in terms of inclusion of black children in clinical trials, by higher mortality and morbidity of black children from several diseases, unequal inclusion of black people in medical faculty or in managerial roles within universities, and lack of consideration of the specific health problems of black children during doctors’ training [4, 5].

The role of physicians in times of social injustice and distress is difficult to navigate. They may inadvertently feed the structural racism that influences access to care, quality of care, and subsequent health disparities. In an effort to engender trust in what they would like to see as a “post-racial” society, some clinicians proclaim that they “do not see color” [6]. However, “color must be seen,” because otherwise, “looking through a racially impervious lens, clinicians neglect the life experiences and historical inequities that shape patients and disease processes” [6, 7]. When dealing with a Black child and his family, this “racially impervious lens” does not allow to make a comprehensive clinical evaluation and think about all differential diagnoses, risking not only to delay the right diagnoses but also to medicalize too much (or not enough) the Black child.

However, our generation of medical doctors is not educated to do otherwise. In fact, during medical schools and residencies, knowledge about health and cultural racial diversity is lacking (Fig. 1, panel A). Knowledge of this diversity can improve the ability of all students, medical doctors under training and therefore specialists to face the needs of an increasingly diverse population of patients.

**Fig. 1:**
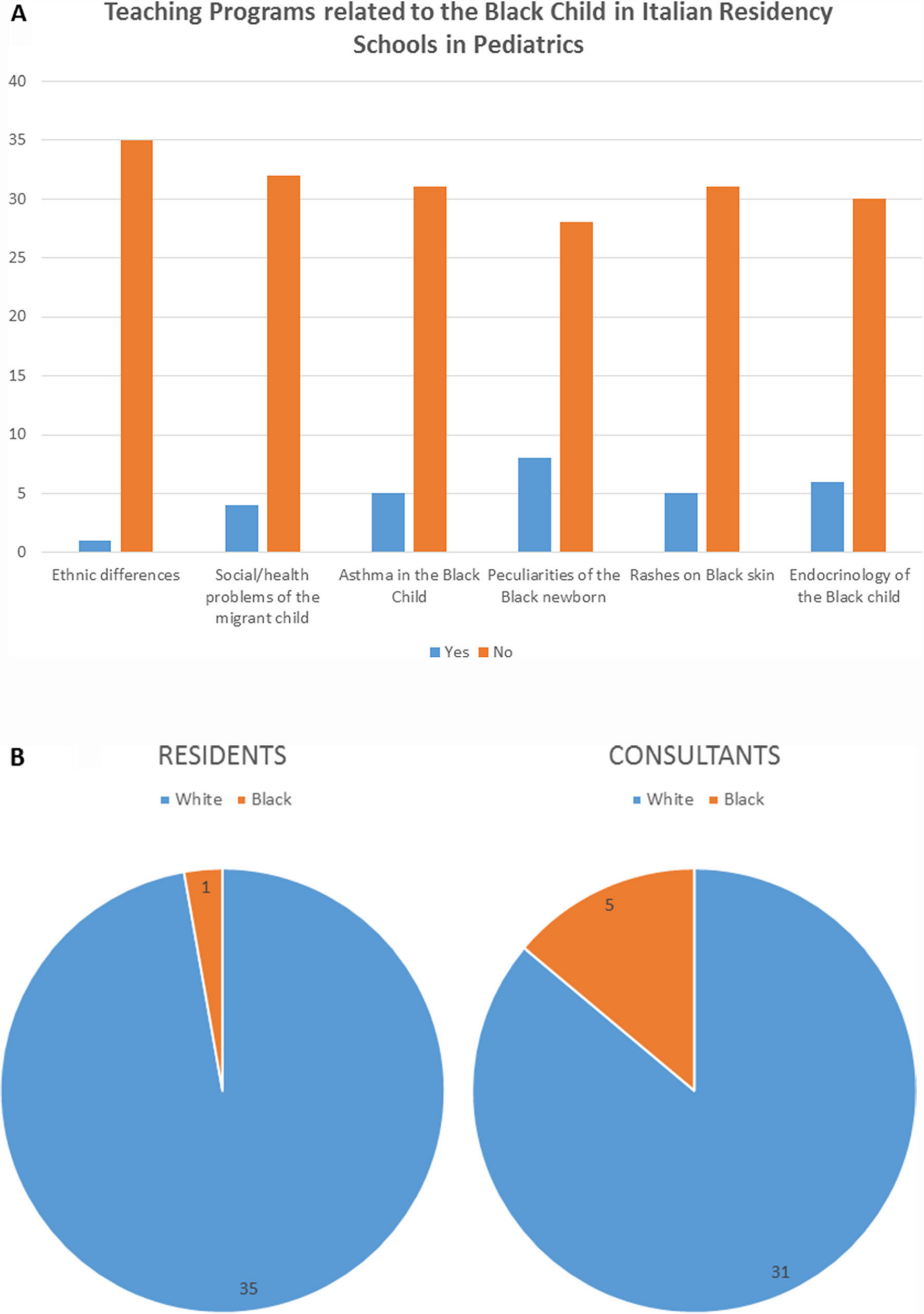
The results showed in the panels are part of a wider national survey aimed to assess discrimination and inequalities in Italian Pediatric Residency Schools. The part 1 of the survey is directed to all residents and aimed to assess their general attitude toward the problematic and impact of racism on human health (not showed and still ongoing, available in Italian on https://www.surveymonkey.com/r/DiscriminazioneInPediatria). Part 2 of the survey was directed to one representative of each of the 36 Italian Residency Schools in Pediatrics in order to assess if teaching programs include specific medical issues related to race/ethnicity (**panel A**) and the presence of Black doctors (**panel B**). Each representative responded, having therefore a response rate of 100%. In particular, in the **panel A** (teaching programs), we assessed the following issues: during your residency school, are there training courses/seminars on cultural/social/religious differences between different ethnic groups? Yes, 2.7%; no, 97.3%; during your residency school, are there training courses/seminars on potential social (immigration, stay in reception centers, stay in refugee camps, abuse and violence) and health problems of the migrant child? Yes, 11.1%; no, 89.9%. During your residency school, have you ever received a specific training on the different frequency, presentation, evolution, and management of asthmatic pathology in the Black child? Yes, 13.9%; no, 86.1%. During your residency school, have you ever received a specific training on common neonatal conditions (jaundice, anemias, low birth weight, maternal-fetal malnutrition, infectious diseases, congenital disorders) that have specific frequency, etiology, presentation, evolution, and management in Black? Yes, 22.2%; no, 77.8%. During your residency school, have you ever received a specific training on the recognition of skin rashes in children with Black skin? Yes, 13.9%; no, 86.1%. During your residency school, have you ever received a specific training on the particular frequency, etiology, presentation, evolution, and management of the endocrinological pathology of the colored child? Yes, 16.7%; no, 83.3%. The **panel B** assessed the presence of Black Residents and Consultants in Pediatric Residence Schools in Italy (2.7% and 13.9% of Residency Schools, respectively). The dataset of responses provided is available upon reasonable request to the corresponding author

Medical journals stressed how racial disparities affect health and medical outcomes [2, 3]; now, it is time for physicians and researchers to act and use their knowledge of human health and disease, including physical and mental health, to create diversity, equity, and inclusion in healthcare.

In the attempt to give our contribution, we share our recent experience by reporting a case that showed us how it is essential to include racial and gender discrimination in a diagnostic process.

On July 15, 2020, a 13-year-old black girl who came to Italy from Niger about 3 years earlier was evaluated in the Pediatric Emergency Department (PED) for sleepiness that had progressively worsened during the last days.

In June, she presented episodes of suspected generalized convulsive seizures, followed by confusion, auditory hallucinations, and drowsiness. She was taken to another hospital where she was hospitalized and several investigations were performed to exclude organic brain diseases: brain imaging and electroencephalography were normal. Furthermore, due to an increase in Creatinine phosphokinase and myoglobin serum levels associated with rhabdomyolysis, to rule out a myopathy, the girl was subjected to electromyography, muscle MRI and biopsy, all negative. Other microbiological, metabolic, and immunological conditions were excluded, and the girl was eventually discharged in good clinical conditions on mid-June 2020.

When she came to our attention, in the night of July 15, the girl was sleepy and hyporesponsive with stable vital signs. The evaluating consultant and resident initially suspected Human African Trypanosomiasis (sleeping sickness), with late-onset neurological involvement, due to the following elements [7]: African origin; living in Italy since only 3 years; neurological involvement characterized by the recent episode of suspected seizures associated with acute rhabdomyolysis; anamnestic data of greatest sleepiness for about a month; acute worsening of drowsiness. Blood tests (whole blood count, blood chemistry tests, inflammatory markers, coagulation screening) were normal. Similarly, brain CT and the search for parasites on blood were negative. The girl was kept under observation and there was discussion about the need to perform other second-level diagnostic tests (such as lumbar puncture, EEG during sleepiness, brain MRI).

Meanwhile, on the next morning, Dr. De Rose, a White-female 4-year resident in pediatrics, began her morning rotation in the PED. Since the patient arrived to Italy in 2017 taking a year-lasting travel from Niger, Dr. De Rose considered the possible traumatic experiences the girl might have gone through during the migratory flow. Therefore, talking to her mother and going in-depth into the personal history of the girl and the whole family, the doctor found out elements that suggested a condition of fragility linked to the family history and the migratory journey. In particular, among others, an important traumatic event for the whole family was the sexual abuses suffered by the older sister during the journey. After this traumatic episode, our patient’s sister developed a condition of clinical depression, and during the previous year, she attempted suicide. She was currently undergoing pharmacological treatment with benzodiazepines, which started about a year earlier.

In light of the traumatic experiences that arose from the story of the patient, made of discriminations, physical and psychological abuses, before taking the second level diagnostic tests, Dr. De Rose asked for an in-depth neuropsychological evaluation. The child psychiatrist, being aware of the whole medical and personal history of the girl and her family and after having done a medical examination of the patient—who was always sleepy but awakenable—confirmed the hypothesis that rather than sleep sickness or another organic disorder, the girl was suffering from a functional disorder.

The risk factors that oriented the child psychiatrist for such a diagnosis were its African origins, the physical and psychological distress experienced by the whole family and linked to the migration, the sister’s sexual abuse and her neuropsychiatric disorder, along with the negative blood and instrumental tests. The case was discussed with the child neuropsychiatrist and sleep-EEG was normal. In the meantime, the toxicological urine analysis was positive for Benzodiazepines.

Therefore, the drowsiness and the acute sleepiness were explained by an improper intake of Benzodiazepines (the same prescribed to the sister for her neuropsychiatric disorder). Instead, as hypothesized by the child neuropsychiatrist, the suspected seizure crisis she presented 1 month earlier could have been explained as a psychogenic nonepileptic seizure belonging to dissociative disorders [8–11] (supplementary material for definitions)*.*

The girl did not undergo other instrumental examinations and her acute clinical conditions improved without pharmacological treatments. The family has been assigned to the local social and neuropsychiatric services for comprehensive support and care.

Why a 4-year female resident managed to establish a specific clinical suspicion and to create a deeper link of trust with the whole family, being able to capture clinical and social elements, that other clinicians have failed to do? I (Dr. Buonsenso) believe the answer lies in her particular sensitivity and personal propensity to the problem of racism and discrimination. I am a consultant in pediatrics and have been supervising her during the last years. Although our initial projects focused, and still do, on point-of-care ultrasound, our interests spontaneously grew and matched on ethnic discriminations and inequalities in child/family health. When I was a resident, I started, and I am currently running, a social and primary health care project in Sierra Leone (West Africa). She is actively involved in volunteering in activities that deal with social, cultural, and health disorders caused by the migratory journeys. I believe that the female White resident saw not only the Black skin of the girl and her family but also the girl (and family) as “someone who already knew,” a story that she recognized. Moreover, this allowed a greater identification with the girl as a woman.

This helped orienting herself in the diagnostic process based not only on the clinic and medical history but also on the patient’s origins and the personal, family, and social background, leading her to the right diagnosis.

In the USA, researchers showed that “in a world still shaped by systemic racism, Black patients are more likely to trust, and heed the advice of, Black physicians” [5, 7, 12, 13]. In the same way, in our history, the White woman resident in the fourth year of our history, with her background and her personal inclination to the subject, has managed to center the problem more easily than other doctors with more professional experience than her.

On the other hand, we realized that not all doctors in Italy, but also in other countries of the world as already described in particular in the USA [5], have the same personal and professional background. They are not used to having in mind the problem of discrimination and inequality, both in personal and professional life.

Following the death of George Floyd, the world’s leading medical journals (The Lancet - https://www.thelancet.com/antiracism - and NEJM - https://www.nejm.org/race-and-medicine) have highlighted serious inequalities and ethnic discrimination in health care.

In Italy, no one has ever systematically evaluated all this, and in particular, no one has evaluated the degree of knowledge acquired during the years of medical training on discrimination and inequalities in health care.

Therefore, starting from our experience and from the data and the reports already reported in the literature in other countries of the world [1-7; 11-13], we decided to create and administer a survey to the doctors in training to evaluate whether in the Italian Paediatric Residency Schools, health and social issues related to different ethnic groups are sufficiently considered.

The preliminary results showed in the panels A and B (Fig. 1) are part of a wider national survey. The part 1 of the survey is directed to all residents and aimed to assess their general attitude toward the problematic and impact of racism on human health (not showed and still ongoing, available in Italian on https://www.surveymonkey.com/r / Discrimination in Pediatrics). The part 2 of the survey was directed to one representative of each of the 36 Italian Residency Schools in Pediatrics in order to assess if teaching programs include specific medical issues related to race/ethnicity (panel A) and the presence of Black doctors (panel B) . Each representative responded, having therefore a response rate of 100%.

In particular, the preliminary results described in panel A (teaching programs) show that during the residency school, in 97.3% of cases, there are no training courses/seminars on cultural/social/religious differences between different ethnic groups; in 89.9% of cases, there are no training courses/seminars on potential social (immigration, stay in reception centers, stay in refugee camps, abuse and violence) and health problems of the migrant child; only 13.9% received specific training on the different frequency, presentation, evolution, and management of asthmatic pathology in the Black child; only 22.2% received a specific training on common neonatal conditions (jaundice, anemias, low birth weight, maternal-fetal malnutrition, infectious diseases, congenital disorders) that have specific frequency, etiology, presentation, evolution, and management in Black; only 13.9% received a specific training on the recognition of skin rashes in children with Black skin; only 16.7% received specific training on the particular frequency, etiology, presentation, evolution, and management of the endocrinological pathology of the colored child.

Furthermore, the preliminary results of the survey shown in panel B (Fig. 1) show that in Italy, Black doctors are rare, so in addition to promoting and involving more Blacks in the medical workforce by removing racial prejudice along the educational path, it is necessary to do something immediately to face the linguistic and cultural barriers between Italian White doctors and Black children and their families.

Increasing trust is challenging; however, the antiracism movements we are seeing on the news can inspire the medical community on reflecting about what means being Black in the Italian or American society. Understanding the Black community historical and cultural background, coupled with improved patient–clinician conversations, could bring Black families closer to the final goal of better access to healthcare and improved quality of life.

Medical ability has been allowing us to respond rapidly to a novel virus in order to save lives. The expertise of doctors and researchers must be used to evaluate this hidden crisis as well, to address racism and injustice, and to protect vulnerable people from harm. This can be done by inviting pediatricians, starting from their educational path, to learn the different forms of discrimination and racism (structural, interpersonal, and internalized); know that life experiences and historical inequities can shape patients and disease processes; examine their own unconscious prejudices by training themselves on the cultural/social/religious differences between the different ethnic groups; optimize education and research to fill the health gap for Black children.

Discrimination and racism should be routinely considered as causative agents or triggers of disease and routinely included in clinical examination, during history collection and evaluation of vital signs. This will benefit child and family health, worldwide.

## Supplementary Information

ESM 1 (DOCX 15 kb)

## Abbreviations

COVID-19: COronaVIrus Disease 19
PED: Paediatric Emergency Department
