# Supplementary material for: Time for Inclusion of Racial and Gender Discrimination in Routine Clinical Assessment
Source: J Racial Ethn Health Disparities. 2021 May 24;8(4):803–8. doi: 10.1007/s40615-021-01061-0 (PMC8143060; doi:10.1007/s40615-021-01061-0)
Supplement: Supplementary file 1 — (DOCX 15 kb) [file 40615_2021_1061_MOESM1_ESM.docx]

**Supplementary material**

***Definition of dissociative disorders and their association with traumatic experiences***

Dissociative disorders are characterized by troubles in the areas of memory, of identity, of emotion, perception, behavior, sensory-motor functioning and of sense of self [1], where nonepileptic seizures have been hypothetized to be a psychoform and somatoform dissociation mechanism where emotions alter executive control affecting motor-function (thus resulting in seizure-like episodes) [2].

The existing association between traumatic experiences and dissociative disorders is now well-established and broadly documented both in children and adult populations, with sexual abuse being the form of maltreatment most predictive of pathological dissociation [3]. This data calls for a specific attention, especially in at-risk populations (such as immigrants) in carefully exploring and identifying the presence of potential maltreatment history in the life of patient so to eventually link it to its somatoform functioning.

**References**

1. American Psychiatric Association. Diagnostic and statistical manual of mental disorders (DSM-5®). *American Psychiatric Pub.* 2013.
2. van der Kruijs SJ, Bodde NM, Vaessen MJ, et al. Functional connectivity of dissociation in patients with psychogenic non-epileptic seizures. *J Neurol Neurosurg Psychiatry*. 2012;**83**(3):239-247.
3. Dorahy MJ, Middleton W, Seager L, Williams M, Chambers R. Child abuse and neglect in complex dissociative disorder, abuse-related chronic PTSD, and mixed psychiatric samples. *J Trauma Dissociation.* 2016;**17**(2):223-236.
